# Cardiovascular Involvement in Autoimmune Diseases

**DOI:** 10.1155/2014/367359

**Published:** 2014-07-22

**Authors:** Jenny Amaya-Amaya, Laura Montoya-Sánchez, Adriana Rojas-Villarraga

**Affiliations:** ^1^Center for Autoimmune Diseases Research (CREA), School of Medicine and Health Sciences, Universidad del Rosario, Carrera 24 No. 63C-69, 11001000 Bogotá, Colombia; ^2^Mederi, Hospital Universitario Mayor, Calle 24 No. 29-45, 11001000 Bogotá, Colombia

## Abstract

Autoimmune diseases (AD) represent a broad spectrum of chronic conditions that may afflict specific target organs or multiple systems with a significant burden on quality of life. These conditions have common mechanisms including genetic and epigenetics factors, gender disparity, environmental triggers, pathophysiological abnormalities, and certain subphenotypes. Atherosclerosis (AT) was once considered to be a degenerative disease that was an inevitable consequence of aging. However, research in the last three decades has shown that AT is not degenerative or inevitable. It is an autoimmune-inflammatory disease associated with infectious and inflammatory factors characterized by lipoprotein metabolism alteration that leads to immune system activation with the consequent proliferation of smooth muscle cells, narrowing arteries, and atheroma formation. Both humoral and cellular immune mechanisms have been proposed to participate in the onset and progression of AT. Several risk factors, known as classic risk factors, have been described. Interestingly, the excessive cardiovascular events observed in patients with ADs are not fully explained by these factors. Several novel risk factors contribute to the development of premature vascular damage. In this review, we discuss our current understanding of how traditional and nontraditional risk factors contribute to pathogenesis of CVD in AD.

## 1. Introduction

Autoimmune diseases (ADs) represent a broad spectrum of chronic conditions that may afflict specific target organs or multiple systems with a significant burden on quality of life. These conditions have common mechanisms including genetic and epigenetic factors, gender disparity, environmental triggers, pathophysiological abnormalities, and certain subphenotypes which are represented by the autoimmune tautology [1–3]. Atherosclerosis (AT) was once considered to be a degenerative disease that was an inevitable consequence of aging. However, research in the last three decades has shown that AT is not degenerative or inevitable. It is an autoimmune-inflammatory disease associated with infectious and inflammatory factors characterized by lipoprotein metabolism alteration that leads to immune system activation with the consequent proliferation of smooth muscle cells, narrowing arteries, and atheroma formation [4]. Both humoral and cellular immune mechanisms have been proposed to participate in the onset and progression of atheromatous lesions [5].

In recent years, many reports have focused on the immunological background of AT, and there is no longer any doubt that it shares several autoimmune pathways [6, 7]. Therefore, it is not surprising to find an accelerated AT in quite a lot of ADs. Several risk factors, known as classic risk factors, have been described since the Framingham heart study. Over time, these lead to endothelial dysfunction, subclinical AT, and cardiovascular (CV) events [8–12]. Interestingly, the excessive CV events observed in patients with ADs are not fully explained by these factors. Several novel risk factors contribute to the development of premature vascular damage. Sarmiento-Monroy et al. [13], based on a model of rheumatoid arthritis (RA), proposed a classification for nontraditional risk factors in ADs, which divided them into genetic determinants, AD-related, and miscellaneous [14, 15]. Therefore, a complex interaction between traditional and disease-specific traits leads to a premature AT process in autoimmunity. All of these pathways may possibly converge into a shared proatherogenic phenotype [16]. While ADs are characterized by a high degree of cardiovascular disease (CVD), there are several subphenotypes such as arterial hypertension (HTN); coronary artery disease (CAD): angina, ischemic heart disease (IHD), and myocardial infarction (MI); congestive heart failure (CHF); peripheral vascular disease (PVD); left ventricular diastolic dysfunction (LVDD); cerebrovascular disease (cerebrovascular accidents (CVAs); transient ischemic attacks (TIAs)); thrombosis: deep vein thrombosis (DVT), pulmonary embolism (PE); and subclinical AT.

In this paper, we discuss our current understanding of how traditional and nontraditional risk factors contribute to pathogenesis of CVD in ADs. It has become evident over the last few years that some ADs are characterized by common pathogenic mechanisms and high rates of morbidity and mortality that are mainly CVD-related. The increased CV mortality in the 3 rheumatic disorders studied the most (i.e., RA, systemic lupus erythematosus (SLE), and antiphospholipid syndrome (APS)) appears to be caused by vascular damage secondary to accelerated AT. However, the burden of CV involvement in other ADs (Sjögren's syndrome (SS) and systemic sclerosis (SSc)) appears to be lower and it is characterized by specific risk factors in addition to those shared with the general population.

## 2. Methods

Studies were identified via a MEDLINE search using the following medical subject heading (MeSH) terms: “Arthritis, Rheumatoid” OR “Lupus Erythematosus, Systemic” OR “Antiphospholipid Syndrome” OR “Sjögren's Syndrome” OR “Scleroderma, Systemic” AND “Cardiovascular Diseases.” Each group was cross-referenced with the following MeSH terms/keywords: “risk factors,” “traditional risk factors,” “classic risk factors,” “nontraditional risk factors,” and “novel risk factors.” Each term was counted for the greatest number of results. Limits regarding language (i.e., English), age (i.e., adults), and humans were taken into account. Assessment for inclusion of studies was done independently by two blinded reviewers (JAA-LMS). Disagreements between them were resolved by consensus using predefined eligibility criteria, from inception up to February 2014.

### 2.1. Study Selection, Data Extraction, and Quality Assessment

Abstracts and full-text articles were reviewed in search of eligible studies. A study was included if (a) the abstract was available, (b) it contained original data, (c) it used accepted classification criteria for each AD, (d) it measured CV risk factors, and (e) it examined clinical endpoints. Articles were excluded from the analysis if they dealt with juvenile pathologies or were done on animal models. Studies were also excluded if they were reviews or case reports, if they discussed topics not related to CVD in AD, if they did not meet the inclusion criteria, if they had insufficient data, or if they had results that showed lack of statistical significance. Likewise, the two blinded reviewers (JAA, LMS) looked for duplicates, excluded them, and organized selected articles. Only novel and classic risk factors [14, 15] with statistical significance were included.

## 3. Results

**Figure 1:**
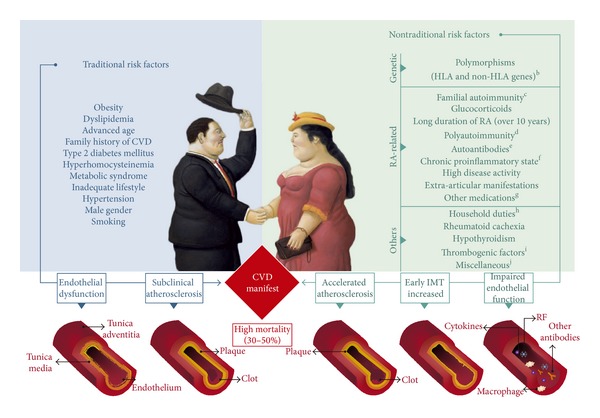
Traditional and nontraditional risk factors for cardiovascular disease in rheumatoid arthritis. AD: autoimmune disease; CVD: cardiovascular disease; IMT: intima-media thickness; RA: rheumatoid arthritis; RF: rheumatoid factor. ^a^CVD includes a broad spectrum of subphenotypes: stroke/transient ischemic attack, coronary artery disease, myocardial infarction, angina, congestive heart failure, arrhythmias, ventricular diastolic dysfunction, hypertension, pulmonary embolism, deep vein thrombosis, and peripheral arterial/venous disease. ^b^Mainly HLA-DRB1∗0404 shared epitope alleles. ^c^The presence of any diagnosed AD in first-degree relatives of proband. ^d^The presence of two concomitant AD in a single patient on the basis of international criteria. ^e^Rheumatoid factor, anti-cyclic citrullinated peptides antibodies, anti-oxidized low-density lipoprotein, anticardiolipins, anti-phosphorylcholine, anti-modified citrullinated vimentin, anti-apolipoprotein A-1, and anti-cytokeratin 18 antibodies. ^f^High levels of c-reactive protein and erythrocyte sedimentation rate. ^g^Methotrexate, leflunomide, and nonsteroidal anti-inflammatory drugs. ^h^Patients (females and males) with RA working on household duties. ^i^von Willebrand factor, plasminogen activator inhibitor-1, and tissue plasminogen activator. ^j^Hypothyroidism, periodontal disease, and other markers such as mannose-binding lectin, serum pentraxin 3, osteopontin, osteoprotegerin, and seric uric acid.

**Figure 2:**
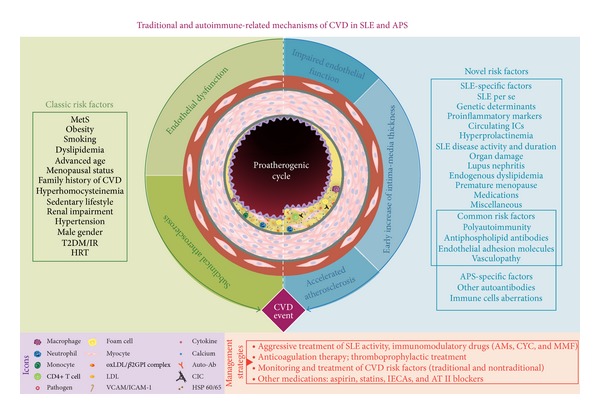
Traditional and autoimmune-related mechanisms of cardiovascular disease in systemic lupus erythematosus and antiphospholipid syndrome. A complex interaction between traditional and disease-specific traits leads to premature atherosclerosis process. Several risk factors (left) have been described since the Framingham heart study, known as classic risk factors, which over time conduce to endothelial dysfunction, subclinical atherosclerosis, and CV event manifest. In the autoimmune setting (right), several novel risk factors contribute to development of premature vascular damage. This damage is represented by impaired endothelial function and early increase of intima-media thickness, which are surrogates of the accelerated atherosclerosis process. These associations are even more pronounced in this case of polyautoimmunity (SLE and APS in the same individual), where risk factors have additive effects and atherosclerosis develops earlier. The cornerstone of management of CV risk includes an aggressive treatment of disease activity, the continuous monitoring and treatment of modifiable CV risk factors, and the use of other medications in order to diminish the CV burden. ACE-I: angiotensin-converting enzyme inhibitors; AMs: antimalarials; APS: antiphospholipid syndrome; AT-II blockers: angiotensin II receptor blockers; Auto-Ab: autoantibodies; AZA: azathioprine; CIC: circulating immune complex; CYC: cyclophosphamide; CVD: cardiovascular disease; HDL: high-density lipoprotein; HRT: hormone replacement therapy; IR: insulin resistance; MetS: metabolic syndrome; MMF: mycophenolate mofetil; oxLDL/*β*2GPI complex: oxidized low-density lipoprotein/2 glycoprotein I; SLE: systemic lupus erythematosus; T2DM: type 2 diabetes mellitus.

There were 6,324 articles identified in PubMed. Of these, 5,800 were identified as duplicates, lacking data or significant statistical associations. A total of 524 full-text articles were assessed for eligibility. Only 322 articles were included for methodological analysis. Finally, 168 articles that had interpretable data and fulfilled the eligibility criteria were included. Several traditional cardiovascular risk factors such as dyslipidemia, hyperhomocysteinemia, smoking, and T2DM had been reported. Many studies were associated with nontraditional risk factors such as genetic markers, autoantibodies, duration of the diseases, markers of chronic inflammation, polyautoimmunity, and familial autoimmunity. These factors and their associations are depicted in Tables 1, 2, 3, 4, and 5 and in Figures 1 and 2.

### 3.1. Rheumatoid Arthritis

A broad spectrum of subphenotypes and mortality due to CVD, including stroke, HTN, IHD, intima-media thickness (IMT), CAD, MI, PVD, thrombosis, and LVDD were described in RA, and the general prevalence range is 30%–50% [17–26].  Table 1 shows the main traditional and nontraditional risk factors associated with CVD in RA, and Figure 1 exemplifies these associations.

### 3.2. Systemic Lupus Erythematosus

CVD is at least doubled among SLE patients compared to other populations and mortality is also increased [27]. CVD burden in SLE includes carotid plaques, MI, angina, CHF, stroke, IMT, PVD, pericarditis, and others discussed below [16, 28–35].  Table 2 shows traditional and nontraditional risk factors associated with CVD in SLE.

### 3.3. Antiphospholipid Syndrome

The prevalence of CVD ranges from 1.7 to 6%, and it could increase up to 14% in patients with antiphospholipid antibodies (APLA). On the other hand, the prevalence of CVD in asymptomatic AT reaches 15% compared to 9% in SLE patients and 3% in normal controls [36, 37]. In the Euro-Phospholipid cohort, MI was the presenting manifestation in 2.8% of the patients, and it appeared during the evolution of the disease in 5.5% of the cohort [38]. Cardiac manifestations may be found in up to 40%, but significant morbidity appears in only 4–6% of these patients. Most of these manifestations are explicable on the basis of thrombotic lesions either in the coronary circulation or on the valves [39].  Table 3 shows the main traditional and nontraditional risk factors associated with CVD in APS.

### 3.4. Sjögren's Syndrome

CV events occurred in 5–7.7% with stroke, MI, CVA, DVT, and arrhythmias [40–44] being the most frequent. Furthermore, tricuspid regurgitation, injured mitral and aortic valves, pulmonary hypertension, and increased left ventricular mass have also been reported [45].  Table 4 shows the main traditional and nontraditional risk factors associated with CVD in SS.

### 3.5. Systemic Sclerosis

A broad spectrum of subphenotypes and mortality due to CVD have been described. Mortality in patients with SSc caused by CVD is between 20 and 30% and, despite being similar to the general population, it occurs a decade earlier (11). CV symptoms are found in 10% of the SSc patients while asymptomatic patients with coronary artery calcification (CAC) accounted for approximately 33.3% in diffuse SSc and 40% in limited SSc [46–54]. However, Doppler results have shown that 64% of the patients have carotid stenosis, compared to 35% of the control patients [55]. Arrythmias, coronary spasm, MI, PVD, CVA, CAD, LVDD, and myocardial fibrosis [46, 52, 54, 56–60] are also defined.  Table 5 shows the main traditional and nontraditional risk factors associated with CVD in SSc.

## 4. Discussion 

This review adds further evidence about high frequency of CVD in patients with ADs and their traditional (i.e., dyslipidemia, abnormal BMI, and male) and nontraditional risk factors (i.e., steroids, household duties, and autoantibodies) [14, 15]. It also highlights the impact on public health and the need to develop new strategies in prediction, prevention, and treatment. Through the review, several factors and outcomes related to CVD were also identified.

### 4.1. Physiopathology of Atherosclerosis in AD

AT is a multifactorial, chronic, and inflammatory disease that had been traditionally viewed as a lipid-based disorder affecting the vessel walls. Nowadays, this theory has been modified, and it is known that all arms of the immune system take part in atheroma formation. The increased understanding of the mechanisms promoting vascular damage has recently led to a sharper focus on proinflammatory pathways, which appear to play a key role in the development and propagation of the disease. Thus, some of the mechanisms that drive atherosclerotic plaque formation, and therefore CVD, are shared with several ADs although each disease may have particular immunological aberrations that provide specific proatherogenic pathways [5–7, 16, 24, 61–68]. This process is characterized by the accumulation of lipid particles, immune cells, autoantibodies, autoantigens, and the multiple production of inflammatory cytokines such as tumor necrosis factor-*α* (e.g.,TNF-*α*). All these components lead to a gradual thickening of the intima layer, thus causing a decrease in elasticity, narrowing of the arterial lumen, reduction of blood flow, plaque rupture, and, finally, the CV event [69, 70]. The systemic inflammatory response that characterizes AT also involves acute-phase reactants such as erythrocyte sedimentation rate (ESR) and c-reactive protein (CRP) [71–75].

Endothelial dysfunction is the first step leading to AT and has been associated with both traditional and nontraditional risk factors related to several ADs. Other factors involved are high concentrations of angiotensin II, increased smooth muscle hypertrophy, peripheral resistance, and oxidation of low-density lipoprotein cholesterol (LDL) as well as elevated plasma homocysteine concentrations and genetic alterations [76–78]. Thus, the different forms of injury increase endothelium adhesiveness for leukocytes or platelets as well as endothelium permeability with the expression of multiple vascular cell adhesion molecules (VCAM), intercellular adhesion molecules-1 (ICAM-1), selectins, and chemokines [4, 79, 80]. In addition to their differentiation, macrophages (M*ϕ*) are associated with upregulation of toll-like receptors, which enhances a cascade of M*ϕ* activation and release of vasoactive molecules such as nitric oxide (NO), reactive oxygen, endothelins, and proteolytic enzymes. All of them lead to the plaque destabilization and the increased risk for rupture [4, 79, 81–83].

T cells, predominantly lymphocyte T helper 1 (Th1), are also recruited to the subendothelial space. Th1 cells dominate over lymphocyte T helper 2 (Th2) as well as their anti-inflammatory mediators (i.e., IL-4, -5, and -10). This kind of reaction is greater in several ADs with a high production of TNF-*α*, IL-2, IL-6, IL-17, and so forth, which, in combination, activates T cells even more and favors smooth muscle cell migration, proliferation, and foam cell formation [16, 61, 84, 85]. Furthermore, activated M*ϕ* express human leukocyte antigen (HLA) II that allows them to present antigens to T lymphocytes. Smooth muscle cells from the lesions also have class II HLA molecules on their surfaces and can also present antigens to T cells such as ox-LDL and heat shock proteins (HSP) 60/65 [4, 61]. The immune regulatory molecule CD40 ligand and its receptor CD40 are expressed by M*ϕ*, T cells, endothelium, and smooth muscle. Both are upregulated in lesions of AT and thus provide further evidence of immune activation [5, 86]. As ox-LDL is a macromolecule with many potential autoantigens, it is possible that antioxidized low-density lipoprotein antibodies (anti-oxLDL) represent a family of autoantibodies against different autoantigens involved in CVD. Thus, the clinical impact of these autoantibodies might vary. However, there are reports showing that elevated anti-oxLDL titers have been detected in patients with early-onset PVD, severe carotid AT, CHF, CAD, MI, and death [87, 88]. This suggests a proatherogenic role for these autoantibodies and supports a key role for them in the progression of AT [87, 89, 90].

Beta-2 glycoprotein-1 (*β*2GPI) is considered to be an autoantigen in APS. Moreover, it is abundantly expressed within the subendothelial regions and in the intima-media layers at the border of atherosclerotic plaque. Both IgM and IgG anti-*β*2GPI levels are elevated in patients with AT and other inflammatory conditions [91]. *β*2GPI is the actual autoantigen for most anticardiolipin antibodies (ACLA), a group of antibodies with procoagulant activity. The association between APLA, AT, and thrombosis can also be seen outside the setting of autoimmunity. Thus, ACLA promote AT by attracting monocytes into the vessel wall and inducing monocyte adherence to endothelial cells. All of this is mediated by adhesion molecules such as ICAM-1, VCAM-1, and E-selectin [7, 92]. The APLA should be considered more than an AT marker since they can enhance AT and are proatherogenic [93, 94]. Likewise, serum from patients with CVD shows a high prevalence of antibodies against HSP60, which mediate lysis of stressed endothelial cells [91, 95, 96].

### 4.2. Rheumatoid Arthritis

In addition to diarthrodial joints, RA can damage virtually any organ thus leading to potential extra-articular manifestations (EAMs). CVD is considered an EAM and represents the major predictor of poor prognosis and the main cause of death in this population [13, 17, 97, 98]. There is evidence that vascular damage accrual begins prior to the diagnosis of RA and accelerates as the disease progresses. RA patients present with endothelial dysfunction and increased subclinical AT compared to age-matched controls [99–101]. Endothelial function, assessed by brachial artery flow-mediated vasodilation, also worsens with disease duration [102]. The CV mortality is higher in RA and life expectancy of patients with RA is three to ten years less than that of the general population [103, 104]. CVD is known to appear earlier and 3.6 times more frequently than in the general population [70, 98, 105]. Thus, CVD is the leading cause of death for RA patients around the world [106, 107]. Currently, IHD secondary to AT is the most prevalent cause of death associated with CVD in RA patients [108]. Almost all mortality studies have been done on populations of European origin, and there is limited information on other ethnic groups. A meta-analysis of 24 RA mortality studies, published between 1970 and 2005, reported a weighted combined all-cause standardized mortality ratio (meta-SMR) of 1.50 with similar increases in mortality risk apparent from the ratios for IHD (meta-SMR 1.59) and for CVA (meta-SMR 1.52) [109]. RA patients with CVD frequently experience “silent” IHD with no symptoms before a sudden cardiac death. Indeed, sudden cardiac deaths are almost twice as common in patients with RA as in the general population [110]. According to the above, the Rochester Epidemiology Project [100] showed that patients with RA had a greater risk of MI than controls of equivalent age and sex. Recently, Sarmiento-Monroy et al. [13] did a systematic literature review of CVD in the Latin American (LA) population. A wide range of prevalence for CVD has been reported (13.8–80.6%) for this population. The highest prevalence was indicated in Puerto Rican patients (55.9%) by Santiago-Casas et al. [111], while for Brazil [112, 113], Colombia [14, 97, 114, 115], and Argentina [116, 117], a similar prevalence was reported (47.4, 35.1, and 30.5%, resp.). However, the mortality in RA patients has been poorly evaluated in this population. Acosta et al. [118] demonstrated a mortality rate of 5.2% in a six-year follow-up. For both, the most frequent cause of death was CVD in 44.7% and 22.2% of the cases, respectively.  Table 1 and Figure 1 give a summary of the main findings related to traditional and nontraditional CVD risk factors in RA patients. In the Colombian population, Amaya-Amaya et al. [14] found that the traditional risk factors including male gender, hypercholesterolemia, and an abnormal body mass index (BMI) were associated with CVD. Nevertheless, the increased prevalence of CV events in RA is not fully explained by these classic risk factors. Both nontraditional RA risk factors and traditional risk factors act together to develop CVD (Figure 1).

Regarding CV risk screening and management, strategies have been developed for the general population and are based on CV risk score calculators such as the Framingham score and the Systematic Coronary Risk Evaluation (SCORE) model, but the accuracy of these models has not been adequately evaluated in inflammatory arthritis [119]. Recent studies have shown that the SCORE underestimates the actual cardiovascular risk of patients with RA. In this regard, a study showed a high frequency of carotid plaques in the group of individuals included in the category of moderate risk according to SCORE risk charts [120]. The major strategy is to develop healthy life styles as a way to maintain control of classical risk factors. Statins can effectively lower total cholesterol in RA patients and significantly improve the rates of CV-related and all-cause mortality when used for primary prevention of vascular events [121, 122]. Similarly, ACE inhibitors and angiotensin II blockers may also have a favorable effect on inflammatory markers and endothelial function in RA [123, 124]. Regarding novel risk factors, it is necessary to establish an adequate management of the disease [19]. The main goal of the treatment should be to reduce the disease activity, and, therefore, decrease the CV burden [124]. Both conventional [125] and biological disease modifying antirheumatic drugs (DMARDs) are used for this purpose. Some studies have shown greater disease control with nonconventional DMARDs such as anti-TNF agents, which lower CRP and IL-6 levels, increase HDL levels, and improve endothelial function [126–129]. Effective treatment may also result in improved physical activity which subsequently leads to a decreased risk of hypertension, obesity, and diabetes, all important determinants of CV disease [127]. The antimalarial (AMs) drugs have been associated with a better CV outcome, enhanced glycemic control, improved lipid profiles, a decreased thrombosis risk, and a reduced probability of developing T2DM in patients with RA [127, 130, 131]. The glucocorticoids (GC) should be used prudently to minimize CV risk secondary to their effects on metabolic parameters and blood pressure. Altogether, there is no clear evidence that low doses of GC contribute significantly to an enhanced CV risk in inflammatory arthritis in contrast to high doses. GCs rapidly and effectively suppress inflammation in RA and their use might be justified for short-term treatment, for example, for “bridging therapy” in the period between initiation and response to DMARD treatment, although the debate does not appear to be settled yet. Therefore, a conservative approach was chosen in which the use of the lowest dose for the shortest period possible was recommended [19, 124, 125, 132]. Reports indicate that anti-TNF is independently associated with a lower CV risk due to the fact that it reduces CV events in young patients by improving the lipid profile, insulin resistance, endothelial function, and aortic compliance and decreasing progression rates of subclinical AT [124, 133–138]. Other biological therapy also produces the same effect. A good example of that was the improvement of endothelial function following rituximab therapy in patients with RA that had been refractory to anti-TNF-alpha drugs [139, 140]. Finally, data about other biologics are conflicting and preliminary; as such, randomized, controlled studies are needed to identify their CV risk reduction role [69, 70].

### 4.3. Systemic Lupus Erythematosus

SLE occurs most often in young women of child-bearing age, the same population that is at the highest relative risk of subclinical AT [141, 142]. Classically, there is a bimodal mortality pattern among SLE patients with an early peak in the first 3 years after diagnosis due to active disease, infections, and nephritis and a second peak with deaths occurring 4–20 years after SLE diagnosis due to CVD as described by Urowitz et al. [143]. Although the overall mortality rate for SLE patients has improved over the past 30 years, mortality due to CVD (i.e., 3–25%) has remained the same [144–146]. There is strong epidemiologic evidence that CVD risk among SLE patients compared to the general population is at least doubled [27]. Carotid plaque is prevalent in 21% of SLE patients under age 35 and in up to 100% of those over age 65 [147]. The increased risk of MI and angina among SLE patients has been well characterized in a number of population-based studies [146, 148–152]. Bengtsson et al. [152] further corroborated these results in their population-based Swedish study where they demonstrated that the risk of CVA and/or MI in the total SLE population was 1.27-fold higher than that in the general population, but among women with SLE aged 40–49, it was 8-fold higher over the 7-year follow-up period. Several research groups have reported prevalence rates in SLE cohorts. In the Systemic Lupus International Collaborating Clinics-Registry for Atherosclerosis (SLICC-RAS) cohort, there were 8 cases of PVD among 1,249 patients during a 2-year period [153]. In the Lupus in Minorities: Nature versus Nurture study (LUMINA), 5.3% of 637 patients developed PVD over a mean follow-up of 4.4 years [154]. In a recent meta-analysis, Schoenfeld et al. [27] showed that epidemiological data strongly support the hypothesis that SLE patients are at an elevated relative risk of CVD. The variability regarding the relative importance of risk factors for CVD among SLE patients in past epidemiological studies is likely due, in part, to different design methods and different patient and comparison groups. Independent predictive risk factors (from multivariate analysis) for CV events have been assessed in five large prospective cohorts of patients with SLE, including the Baltimore [155], Pittsburg [149], LUMINA [32], Toronto [156], and SLICC-RAS [153] cohorts. The main results are discussed in Table 2 and Figure 2. Diverse SLE cohorts have shown the influence of advanced age, dyslipidemia, obesity, HTN, and hyperhomocysteinemia as classical risk factors for CVD in the lupus population [27, 157–159]. There is strong epidemiological evidence that traditional CVD risk factors also elevate CVD risk among SLE patients (Figure 2). Amaya-Amaya et al. [160] recently added further evidence of the high frequency of CVD in 310 consecutive patients with SLE (36.5%). Their findings on traditional risk factors (i.e., dyslipidemia, smoking), plus the confirmation that coffee consumption is another risk factor, showed that, in combination, they contribute to this complication in the LA population. It is well known that while traditional CVD risk factors are undoubtedly important in increasing the CVD risk among SLE patients, these do not fully account for the elevated risk of CVD in this population. Esdaile et al. [161] evaluated risk factors for CAD in two Canadian lupus cohorts by means of the Framingham multiple logistic regression model and found a high risk of developing CAD after removing the influence of these risk factors. Therefore, SLE-associated factors play an important role in the premature AT process characteristic of those patients [70, 162–166]. Hence, there is an increasing interest in identifying novel risk factors that might explain the development of accelerated AT in these populations. The proposal has been made that SLE be managed the same way that T2DM is—as a “CVD equivalent”—with lower lipid goals, more aggressive aspirin use, and potentially more aggressive monitoring [167, 168].

Recent studies have started to address the question of whether traditional treatment regimens may prevent or slow AT in SLE patients [142]. There are several new mechanisms of action described for AMs, many of which have beneficial effects in the management of CV risk in patients with SLE [131, 169]. There is evidence that AM drugs reduce LDL levels, elevate HDL, and, when taken concomitantly with steroids, can reduce TC [170]. In addition, beneficial effects of HCQ on thrombosis formation have also been described [171–174]. Ruiz-Irastorza et al. [175, 176] found that HCQ use conferred a 50–60% decrease in the risk of CVD. Otherwise, the recent randomized controlled Lupus Atherosclerosis Prevention Study by Petri et al. [28] suggests that atorvastatin did not in fact slow progression of subclinical AT in 200 SLE patients over 2 years. However, in other studies, it has been demonstrated that statins do reduce CD40 levels in vivo and in vitro and, therefore, interfere with CD40-CD40 ligand interactions in both SLE and AT [177]. As inflammation is one of the targets of therapy in SLE, several other immunosuppressant drugs and biological therapies currently employed in SLE could also be considered such as potential new antiatherogenic agents [178, 179].

### 4.4. Antiphospholipid Syndrome

The APS is a prothrombotic state that can affect both the venous and arterial circulations. The deep veins of the lower limbs and cerebral arterial circulation are the most common sites of venous and arterial thrombosis, respectively [180]. The heterogeneity of APS clinical manifestations is likely linked to the varied effects that APLA can induce on endothelial cells [181]. Thrombotic events are the clinical hallmark of APS, occurring in venous and arterial circulations with a high recurrence rate of arterial involvement. They can be expressed as carotid disease, CVA, CAD, and PVD due to thrombus formation or AT [182–188]. Further, other cardiac manifestations may include irregular thickening of the valve leaflets due to deposition of immune complexes that may lead to vegetation and valve dysfunction, which are frequent and may be a significant risk factor for stroke [189–192].  Table 3 and Figure 2 show the main traditional and nontraditional risk factors associated with APS and CVD. Early diagnosis of APS through examination of the heart and aggressive control of all traditional risk factors through lifestyle modifications and pharmacotherapy, probably anti-inflammatory treatment, and close follow-up of APS patients may help to minimize CV risk in these individuals [189, 193]. The APS coagulopathy in these patients requires careful and judicious use of appropriate antiaggregant and anticoagulant therapy [39]. Specifically targeted therapies that exert anti-inflammatory or immunomodulatory effects become important therapeutic tools in APS. In order to achieve beneficial effects, these drugs should primarily antagonize the pathogenic effects of APLA. Moreover, these treatments should also control atheroma, which is one of the major causes of CV mortality in this pathology [177]. For instance, AM drugs may exert evident antiatherogenic properties [168, 194]. Statins also have pleiotropic characteristics, which include antiatherosclerotic (i.e., preventing endothelial dysfunction), anti-inflammatory (i.e., reducing CRP levels), antioxidant, immunomodulatory, and antithrombotic effects [195–200]. Likewise, aspirin has been used in primary and secondary prevention in APS patients particularly for its inhibitory effects on platelet aggregation [201, 202]. In addition to their anticoagulant effects, unfractionated heparins and low molecular weight heparins also have anti-inflammatory properties. Thus, heparins may represent another anti-inflammatory therapeutic tool even though the mechanisms of action responsible for their anti-inflammatory effects are not yet fully understood [203]. Recent improvements in the understanding of the pathogenic mechanisms have led to the identification of novel potential targets and therapies that might be used as new potential immunomodulatory approaches in APS and CVD such as B-cell targeted therapies, complement inhibition, inhibition of costimulation, intracellular pathway inhibition, and anticytokine therapies [204].

### 4.5. Sjögren's Syndrome

This is an autoimmune epithelitis that affects the exocrine glands with a functional impairment that usually presents as persistent dryness of the eyes and mouth [205, 206]. Its clinical spectrum extends from an autoimmune exocrinopathy to a systemic involvement with vasculitis and diverse extraglandular systemic manifestations (40–50%). This includes CVD although with lower prevalence as mentioned above [207, 208]. Chronic systemic inflammation is a risk factor for developing AT, however, and contrary to what is expected, the prevalence of CVD associated with AT is not appreciably increased in patients with SS. This probably is characterized by chronic but milder inflammation as Ramos-Casals et al. showed [205]. In fact, Akyel et al. [209] found endothelial dysfunction in SS patients although their carotid IMT was comparable to the healthy control group. It should be noted that the CV risk in patients with SS is rising as a result of the population affected by the disease (i.e., postmenopausal women) [43, 210]. Vaudo et al. [211] found a high rate of subclinical AT due to changes in the carotid arterial wall studied/seen by femoral and carotid ultrasonography. All these findings (i.e., Table 4) suggest that a functional impairment of the arterial wall may sustain early phases of atherosclerotic damage in SS. A combined effect of disease-related chronic inflammatory and immunological factors appears to support dysfunction of endothelium and vascular smooth muscle cells, respectively.  Table 4 contains the most frequent traditional and nontraditional risk factors related to CVD and SS. The management of CVD in SS patients must be directed toward rigorous intervention of modifiable risk factors as well as nontraditional risk factors, warranting a routine evaluation of autoantibodies and other SS-related factors. Pérez-De-Lis et al. [210] found a protective role of AMs in CVD and SS patients since these drugs show an association with a lower frequency of HTN, T2DM, and dyslipidemia. So, in the future, it will be necessary to analyze the incidence of CVD and the role of the different risk factors listed in Table 4 prospectively for the development of such complications.

### 4.6. Systemic Sclerosis

There are two major disease presentations: the microvascular and macrovascular involvement. The vasculopathy of SSc typically affects the small arteries and capillaries (i.e., microvascular occlusive disease with vasospasm and intimal proliferation) while macrovascular disease has been demonstrated by carotid ultrasonography, ankle brachial blood pressure index, and peripheral angiography [48, 50, 52] due to fibrosis, thickening, and chronic proliferation of the intimal layer as well as transmural lymphocytic infiltrate without evidence of atherosclerotic plaque [48, 53]. However, recently, the evidence has demonstrated increased atherosclerosis, including CAC, higher prevalence of subclinical CAD, and higher carotid IMT [46, 212]. Patchy fibrosis is the most important feature in the myocardium, especially when it is localized in subendocardial regions. This fibrosis usually accompanies LVDD [59, 60], but it is symptomatic in 10% of the cases [213]. There have been reported MI or myocardial perfusion defects with coronary arteries which suggests that the etiology of infarction may be due to microvascular disease rather than coronary AT although we must recognize that the latter is higher in patients with SSc [214, 215]. Patients with SSc have a reduced coronary flow reserve [216, 217], which is associated with higher coronary events [218, 219]. Other authors have reported ectasia, spasm, and coronary artery stenosis [56, 57]. Arrhythmias and conduction disturbances are characteristic of cardiac involvement in SSc as hypertrophy and heart failure contractility [58, 60] have been reported. Ultrasonography evaluation is also used to evaluate the carotid arteries and has been proven to be a useful marker for the assessment of subclinical AT and a strong predictor of subsequent MI and CVA [77, 216, 220]. In addition, once SSc has been diagnosed and established, attention to treatment of the vascular component is critical. While the traditional approach has been solely to use vasodilator therapy, new investigations are underway to develop novel therapies, to prevent further vascular injury, and to stimulate vascular repair. Some of the current treatment approaches include the following: prostacyclin analogs, endothelin antagonists, phosphodiesterase inhibitors, immunosuppressive therapy, and tyrosine kinase inhibitors [221].

### 4.7. Spondyloarthropathies

Since spondyloarthropathies are also chronic autoimmune-autoinflammatory diseases associated with accelerated atherosclerosis, the patients with spondyloarthropathies also have a higher risk of cardiovascular disease than the general population. Ankylosing spondylitis has been associated with increased mortality rate compared to the general population, which is, in great part, the result of cardiovascular complications. Also, subclinical atherosclerosis, manifested by the presence of endothelial dysfunction and increased carotid intima-media wall thickness and carotid plaques, has been observed in patients with psoriatic arthritis and ankylosing spondylitis. In patients with ankylosing spondylitis, TNF-alpha blockade was associated with improvement of insulin resistance, markers of metabolic syndrome, and biomarkers of endothelial dysfunction [222–232].

## 5. Conclusions

AT and ADs share several mechanisms. The excessive CV events observed in patients with ADs are not fully explained by classic risk factors. Several novel risk factors contribute to development of premature vascular damage. Therefore, a complex interaction between traditional and disease-specific traits converges into a shared proatherogenic phenotype in this population. Until additional research and disease-specific risk prediction tools are available, current evidence supports aggressive treatment of disease activity and careful screening for and management of modifiable traditional risk factors in patients with ADs. The finding and understanding of complex interactions between predisposing factors (i.e., genetic, environmental factors, and ADs per se) will allow us to better describe and assess the broad spectrum of CV subphenotypes in ADs and their treatments.

## Figures and Tables

**Table 1:** Traditional and nontraditional risk factors associated with CVD and RA.

Risk factor		Comments	References
Traditional risk factors			
Obesity		(i) Insulin resistance due to release of inflammatory cytokines such as TNF-*α*. (ii) Increased coronary calcification due to insulin resistance. (iii) ↑ Abdominal fat.	[14, 233, 234]
Dyslipidemia		(i) ↓ HDL and ↑ LDL and TAG. (ii) Induces higher risk of IHD.	[14, 19, 97, 233–238]
Advanced age		(i) Old age prompts structural and functional deterioration in the heart and vessels structure. (ii) Senescent immune system is normally associated with phenotypical and functional changes.	[233, 239]
Family history of CVD		Heritable factors: HTN and familial hypercholesterolemia.	[97, 240, 241]
T2DM		(i) Coexistence of T2DM and RA increases three times the risk of developing CVD. (ii) Abdominal obesity, antihypertensive medication, disease activity, and use of GCs affect glucose metabolism in RA patients.	[14, 242, 243]
Hyperhomocysteinemia		(i) It is considered as biomarker for AT and a risk factor related to CAD and CVA. (ii) There is still controversy about whether hyperhomocysteinemia is a causative agent of cardiovascular damage or only an epiphenomenon of inflammation. (iii) A high prevalence of this biomarker had a statistical association with male gender and higher radiological damage.	[235, 236, 244–248]
Metabolic syndrome		(i) Alteration in the production of cytokines and proinflammatory adipokines leads to an increasing activity of RA and an accelerating AT. (ii) It was related to pain and functional status, suggesting disease activity (iii) Increased prevalence of waist circumference, blood pressure, and fasting glucose (i.e., worse prognosis). (iv) Increased epicardial adipose tissue volume.	[103, 236, 242, 247, 249–252]
Sedentary lifestyle		(i) Patients are less physically active than controls due to pain, stiffness, deformity, and impaired mobility. (ii) Impairment of altered lipid pattern.	[97, 252, 253]
Hypertension		Increases the risk of IHD and CVA with important impact on mortality.	[249, 254, 255]
Male gender		Cardiovascular disease is more frequent in male gender.	[14, 254, 256–260]
Smoking		(i) Smokers with RA have worse prognosis than nonsmokers RA patients in terms of RF titers, disability, radiological damage, CVD, and treatment response. (ii) Premature CVD mortality.	[249, 261, 262]

Nontraditional risk factors			
Genetic	HLA-DRB1 SE	(i) Its alleles are related to chronic inflammation, high disease activity, EAMs, endothelial dysfunction, increasing CV events, AT plaque, and premature mortality. Some of them are independent of autoantibody status. (ii) Being a carrier of a single copy of HLA-DRB1 SE was significantly associated with an increased risk of atherosclerotic plaque in RA Colombian patients.	[97, 145, 262–268]
	Non-HLA	(i) Polymorphisms in *endothelin*-*1, MTH*-*FR, TRAF1/C5, STAT4, factor XIIIA, PAI*-*1, TNFR*-*II, LT*-*A, LGALS2, TGF*-*β, GSTT1, ACP1, and NF*-*κβ1 *genes may be contributed to CVD risk and adverse outcome. (ii) Interaction between smoking and polymorphism in the *VEGFA* gene is associated with IHD and MI in RA patients. (iii) The *IL6-174* gene polymorphism may play a role in the development of subclinical atherosclerosis in patients with RA. (iv) *TNFA* rs1800629 (G>A) gene polymorphism is associated with predisposition to CV complications in RA patients. This predisposition seems to be restricted to individuals carrying the SE. (v) Genetically determined high serum levels of MBL and high serum levels of agalactosyl IgG are associated with increased risk of IHD, MI, and premature death.	[78, 97, 269–286]

	RA per se	(i) Independent factor for developing MI and accelerated AT. (ii) It represents a broad spectrum of conditions related with the autoimmune nature of the disease.	[14, 19, 287]
	Familial autoimmunity	(i) It confers additional susceptibility to CVD in RA patients, as well as presence of atherosclerotic plaque, radiographic progression, high disease activity, and persistent inflammation. (ii) Increased frequency of HLA-DR4.	[14, 97]
	Glucocorticoids	(i) It targets inflammation but its adverse effects include carotid plaques, arterial stiffness, decreased insulin sensitivity, elevated lipid levels, hypertension, and CVD. (ii) Patients that are treated with a daily dose >7.5 mg/day appeared to have twice as the risk of heart disease as patients that are in nonsteroidal treatment. (iii) The increased mortality in patients under low-dose oral GC for more than 10 years has been related mainly to CVD.	[14, 19, 111, 124, 240, 288–294]
	Long duration of disease	(i) Disease duration over 10 years was significantly associated with increased risk of atherosclerotic plaque in Colombian population. (ii) Patients with prolonged RA have more atherosclerosis than patients of the same age with more recent disease onset. They have more extensive subclinical atherosclerosis or CAC, independent of other CHD risk factors. (iii) RA duration is independently associated with LVDD suggesting the impact of chronic autoimmune inflammation on myocardial function.	[97, 102, 240, 290, 295–298]
	Polyautoimmunity	It was associated with CVD in Colombian population.	[299]
RA-associated	Autoantibodies	(i) Immune complexes from RF can be deposited in the endothelium generating endothelial dysfunction and AT through inflammatory reactions. (ii) RF-positive patients were at increased risk of CV events following exposure to GC. (iii) RF titers were independently predictive of endothelial dysfunction and increased mortality in RA. (iv) Anti-CCP and RF-IgM were related to impaired endothelial function independent of other CV risk factors, and they are independently associated with impaired left ventricular relaxation and development of IHD. (v) Anti-ox-LDL, ACLA, APLA, and anti-ApoA-1 are associated with early atherosclerotic changes and future thrombotic events. (vi) The presence of ACLA and an altered lipid profile may represent an important risk factor for thrombotic events in patients affected by RA. Anti-PC, anti-HSP 60/65, and anti-MDA-LDL may have independent roles in subclinical AT. (vii) Anti-ox-LDL was strongly related with the degree of inflammation and carotid plaque and may predispose to a higher risk for CVD, as they were independently associated with subclinical atherosclerosis. (viii) High levels of anti-MCV and LDL-immune complexes are risk factors for increased AT and are associated with inflammation.	[9, 97, 238, 299–314]
	Chronic proinflammatory state	(i) It may accelerate atherogenic processes and microvascular dysfunction: accentuation of known pathways of plaque formation. (ii) Inflammatory stimuli may be involved in the initiation of CHF among patients with RA. (iii) Markers of chronic inflammation (i.e., current and cumulative inflammation) such as CRP, ESR, TNF-*α*, IL-6, IL-17, and haptoglobin are present in endothelial activation and increased in carotid IMT, carotid plaque, CAD, CV complications, and mortality. (iv) Both established CV risk factors and manifestations of RA inflammation contribute significantly to carotid atherosclerosis in RA and may modify one another's effects.	[8, 24, 73, 75, 99, 260, 300, 315–319]
	High disease activity	(i) Higher activity index is associated with CV events and mortality. (ii) DAS-28 was a significant predictor of major adverse CV events and mortality. (iii) The occurrence of new CV events in very early RA was explained by traditional CV risk factors and was potentiated by high disease activity.	[97, 268, 300, 316, 320, 321]
	EAMs	(i) Increases three times the risk of having CVD and these patients, also present greater IMT. (ii) CVD is considered a severe EAM of the disease. (iii) Severe EAM manifestations are associated with an increased risk of CVD events. Systemic EAM disease is a major determinant of CVD morbidity.	[145, 240, 266, 296, 322–324]

	Household duties	Employed women are somewhat less physically disabled than their unemployed counterpart (including housework).	[14, 325, 326]
	Hypothyroidism	Fourfold higher risk of CVD even after adjustment for other traditional CV risk factors.	[241, 327, 328]
Others	Thrombogenic and other factors	(i) State of hypofibrinolysis is associated with CVD progression and levels of von Willebrand factor, PAI-1, and tissue type plasminogen (ii) Other biomarkers have been related to CVD: OPG, OPN, sPTX-3, periodontal disease, hepcidin, seric uric acid, para-articular bone loss, and MBL.	[254, 289, 297, 311, 329–341]
	Rheumatoid cachexia	Associated with high levels of LDL, low levels of atheroprotective anti-PC, and high frequency of HTN in RA patients. Patients with RA experience a 4.3% increase in body fat mass for a given BMI compared to healthy individuals.	[24, 336, 342, 343]

ACP1: acid phosphatase locus; anti-ApoA-1: anti-apolipoprotein A-1 antibodies; ACLA: anticardiolipins antibodies; anti-*β*2GPI: anti-*β*2 glycoprotein I antibodies; anti-CCP: anti-cyclic citrullinated peptide antibodies; anti-HSP: anti-heat shock proteins antibodies; anti-MCV: anti-modified citrullinated vimentin antibodies; anti-MDA-LDL: anti-malondialdehyde modified LDL antibodies; anti-oxLDL: anti-oxidized low-density lipoprotein antibodies; APLA: antiphospholipid antibodies; AT: atherosclerosis; BMI: body mass index; CAC: coronary artery calcification; CAD: coronary artery disease; anti-PC: anti-phosphorylcholine antibodies; CRP: c-reactive protein; CV: cardiovascular; CVA: cerebrovascular accident; CVD: cardiovascular disease; DAS: disease activity index; EAM: extra-articular manifestations; ESR: erythrocyte sedimentation rate; GCs: glucocorticoids; GSTT-1: glutathione S-transferase, HDL: high-density lipoprotein; HTN: hypertension; IHD: ischemic heart disease; IMT: intima-media thickness; LDL: low-density lipoprotein; LGALS2: galectin-2; MBL: mannose-binding lectin; MI: myocardial infarction; LT-A: lymphotoxin-A; MTH-FR: methylene tetrahydrofolate reductase; NF*κ*B1: nuclear factor of kappa light polypeptide gene enhancer in B-cells 1; NO: nitric oxide; OPG: osteoprotegerin; OPN: osteopontin; PAI-1: plasminogen activator inhibitor type-1; IL6: interleukin 6; activator inhibitor type-1; RA: rheumatoid arthritis; RF: rheumatoid factor; SE: shared epitope; sPTX-3: serum pentraxin-3; STAT4: signal transducer and activator of transcription 4; T2DM: type 2 diabetes mellitus; TAG: triglycerides; TGF-*β*1: transforming growth factor beta; TNF-*α*: tumor necrosis factor-*α*; TNFR-II: tumor necrosis factor receptor II; TRAF1/C5: TNF receptor-associated factor 1; VEGF-A: vascular endothelial growth factor A.

**Table 2:** Traditional and nontraditional risk factors associated with CVD and SLE.

Risk factor		Comments	References
Traditional risk factors			
Hypertension		(i) It is more frequent among SLE patients than people with noninflammatory disorders (ii) It acts as CVD subphenotype as well as a risk factor and also influences the risk of death by CVD. It increases the risk of thrombosis and it is more prevalent among SLE patients with atherosclerotic plaque. (iii) Lupus patients with abnormal myocardial scintigraphic findings and hypertension, as risk factor for CAD, had a higher risk of abnormal findings on coronary angiography.	[32, 152, 344–360]
T2DM		(i) T2DM has influence on abnormal myocardial perfusion in asymptomatic patients with SLE. (ii) Alterations in glycemic profile were associated with traditional risk factors for CHD and lupus characteristics, including CVD, damage index, and renal involvement. (iii) Patients with SLE and T2DM were at increased risk of thrombosis, atherosclerotic plaque, and CAC. This risk remains elevated throughout the course of the disease.	[32, 252, 349–352, 357, 358, 361, 362]
Dyslipidemia		(i) The main risk factor for death in SLE was heart involvement, which was influenced by dyslipidemia. The inflammatory context of SLE leads to dysregulation of lipid metabolism pathways → increased risk of atherosclerotic disease and thrombotic events. (ii) Alterations in lipid profile were a risk factor for endothelial dysfunction, myocardial perfusion abnormalities, and premature CAC and CAD in young women.	[252, 344, 345, 350–352, 354, 356, 357, 363–369]
Male gender		(i) Male gender was a risk factor for developing severe organ damage (CVD) and mortality in SLE patients. (ii) Males with SLE were at increased risk of thrombosis and CAC. This risk remains elevated throughout the course of the disease. (iii) Patients had more peripheral vascular and gonadal involvement.	[32, 350, 351, 357, 361, 367, 370, 371]
Metabolic syndrome		(i) SLE patients had a high prevalence of MetS that directly contributes to increasing inflammatory status and oxidative stress. (ii) MetS were associated with traditional risk factors for CAD and lupus characteristics, including CVD, damage Index, and renal involvement. (iii) HCQ use proved to be protective against MetS. (iv) Insulin sensitivity and intima-media thickness are altered in SLE patients, especially those with MetS comorbidity with an associated increase in disease activity and damage (v) Renal lupus, higher corticosteroid doses, Korean and Hispanic ethnicity are associated with MetS in SLE patients	[252, 358, 359, 372–377]
Obesity		(i) Patients with SLE who had excess weight present distinct clinical-laboratory findings, sociodemographic characteristics, and treatment options when compared to normal weight patients. Excess weight is associated with SLE poor prognosis. (ii) Increased weight has influence on abnormal myocardial perfusion in asymptomatic SLE patients. (iii) SLE patients with high BMI have increased QT interval parameters, presence of CAD, and carotid plaque. This prolongation may lead to an increased CV risk.	[32, 252, 345, 349, 352, 357, 358, 369, 378–380]
Smoking		(i) Smoking is an important determinant in the occurrence of thrombotic (central and/or peripheral, arterial and/or venous) events in SLE patients, due to atherosclerotic plaque and thrombosis (ii) Smoking habits influence abnormal myocardial perfusion in asymptomatic SLE patients. (iii) Smoking was a risk factor for premature CAC and CAD in young women with SLE.	[252, 345, 350–352, 354, 357, 358, 370, 372, 381, 382]
Advanced age		Several traditional risk factors, including age, appear to be important contributors to atherosclerotic CV damage.	[349, 352, 361, 383, 384]
Menopausal status		(i) High percentage of SLE patients with abnormal angiographic findings was in postmenopausal status. (ii) There is high prevalence of premature menopausal status as risk factor for CVD. (iii) Postmenopausal status was a risk factor for premature CAC in young women with SLE. (iv) Postmenopausal women had a higher prevalence of subclinical AT and abnormal myocardial perfusion in asymptomatic patients with SLE.	[351, 352, 354, 356–358, 367, 385, 386]
Family history of CVD		(i) Familial history of CVD was an independent risk factor for atherosclerotic process and premature CAC in women with SLE. (ii) Family history of CVD influences abnormal myocardial perfusion in asymptomatic SLE.	[32, 351, 352, 354, 357, 358]
HRT		HRT use was not associated with the occurrence of vascular arterial events in the LUMINA patients. HRT use in women with SLE should be individualized, but data suggest its use may be safe if APLA are not present or vascular arterial events have not previously occurred.	[32]
Hyperhomocysteinemia		(i) Hyperhomocysteinemia was a risk factor for CAC in SLE patients. (ii) The presence of polyautoimmunity and hyperhomocysteinemia was a risk factor for thrombotic events.	[351, 387]

Nontraditional risk factors			
Genetic determinants	Ancestry	There are several differences regarding clinical (including CVD), prognostic, socioeconomic, educational, and access to medical care features in GLADEL cohort according to ancestry (White, Mestizo, and African-LA).	[15, 360, 388]
	Non-HLA	(i) A SNP in *FGG* rs2066865 demonstrated association with arterial thrombosis risk in Hispanic American patients with SLE. (ii) The *CRP GT20* variant is more likely to occur in African-American and Hispanic SLE patients than in Caucasian ones, and SLE patients carrying the GT20 allele are more likely to develop vascular arterial events (LUMINA multiethnic cohort). (iii) *TRAF3IP2* may affect disease phenotype and, particularly, the occurrence of pericarditis. (iv) There is a considerable genetic component for CAD with *IRF8* as a strong susceptibility locus.	[382, 389–391]

	Polyautoimmunity	(i) The presence of APS and its characteristic antibodies was the major independent contributor to the development of thrombotic events and severe organ damage. (ii) Polyautoimunity (e.g., APS) may suggest concerted pathogenic actions with other autoantibodies in the development of thrombotic events.	[3, 15, 353, 392–394]
	SLE per se	(i) SLE diagnosis is associated with carotid plaque formation and development of CV event. (ii) High percentage of patients with abnormal angiographic findings had higher ACR criteria number for SLE. (iii) Endothelial dysfunction is associated with traditional and SLE-specific risk factors, and early data suggest reversibility of endothelial dysfunction with therapy.	[34, 356, 369, 388]
	Autoantibodies	(i) One of the independent predictors of vascular events in a multiethnic US cohort (LUMINA) was the presence of any APLA. (ii) Anti-*β*2GPI antibodies were strongly associated with thrombosis. The decrease of anti-*β*2GPI levels at the time of thrombosis may indicate a pathogenic role.	[32, 365, 371, 392, 395–398]
		(iii) The higher frequency of aPT found in thrombosis may suggest concerted pathogenic actions with other autoantibodies in the development of thrombotic events. (iv) Patients with ACLA seem to be at an increased risk for arterial and venous thrombotic events and showed an association with echocardiographic abnormalities. (v) There was correlation between lupus anticoagulant and thrombotic events in Brazilian lupus patients.	
	Immune cells aberrations	(i) Complement fixing activity of ACLA seems to be relevant in thrombotic venous events. (ii) Activation of endothelial MMP-2 by MMP-9 contained in NETs as an important player in endothelial dysfunction and MMP-9 as a novel self-antigen in SLE. These results further support that aberrant NET formation plays pathogenic roles in SLE.	[393, 399]
	Inflammatory markers	(i) Increased ESR and CRP were independently associated with MetS and vascular events in lupus patients.	[32, 361, 373]
	Endogenous dyslipidemia	(i) HDL distribution and composition (−HDL2b, +HDL3b, and +HDL3c) were abnormal in SLE patients. (ii) Low HDL levels and increased TAG levels were associated with AT by cIMT measurement. (iii) SLE pattern of dyslipoproteinemia may increase the risk of developing CAD.	[400–402]
SLE-associated	Disease activity	(i) Disease activity (SLAM) is an important determinant in the occurrence of thrombotic (central and/or peripheral, arterial and/or venous) events in the LUMINA cohort. (ii) SLEDAI scores were positively correlated with abnormal BMI and WC. (iii) Higher disease activity (i.e., SLEDAI and SLICC) is a predictor of CAC and it was independently associated with MetS, myocardial perfusion abnormalities, and thrombosis. Higher score of SDI was associated with atherosclerotic plaque in Brazilian SLE patients. (iv) SLE patients have a lipid profile abnormality which is aggravated by disease activity and may reside in a defect of VLDL metabolism. (v) There is a close link between MeTS and SLICC/ACR score with increased aortic stiffness.	[350, 351, 356, 369, 372, 373, 381, 402–404]
	Organ damage	(i) Baseline and accrued damage increase mortality risk (including due to CVD). (ii) Measured by SDI, patients had more peripheral vascular involvement. (iii) MetS was associated both with traditional risk factors for CHD and with lupus characteristics including damage index. (iv) There was a correlation between IMT and revised damage index (SLICC). (v) Atherosclerotic CV damage in SLE is multifactorial, and disease-related factors (including CRP levels and SDI at baseline) appear to be important contributors to such an occurrence.	[358, 361, 369, 371, 405, 406]
	Long duration	(i) Longer duration of SLE was associated with atherosclerotic plaque and CV events. (ii) A correlation between IMT and duration of the disease was found in SLE patients. (iii) Disease duration was an independent predictor for premature CAC in young women with SLE.	[352, 354, 369, 383]
	Medications	(i) PDN >10 mg/day was independently associated with MetS and IMT in SLE patients. (ii) IHD was observed in SLE patients: those with long term steroid therapy and those with frank episodes of vasculitis.	[352, 355, 373]
	Vasculopathy	(i) Current vasculitis was associated with abnormal myocardial scintigraphy. (ii) Patients with SLE and RP seem to be at increased risk for arterial and venous thrombotic events. IHD was observed in SLE patients: those with long term steroid therapy and those with frank episodes of vasculitis.	[355, 357, 396]
	Renal involvement	MetS were associated with traditional risk factors for CHD and lupus characteristics, including damage index and renal involvement (nephritic syndrome).	[358]

	BMD	Decreased BMD was an independent predictor for premature CAC.	[354]
Miscellaneous	Sociodemographic factors	A low education and monthly income were associated with MetS.	[252]
	25(OH) levels	Lower baseline 25(OH) vitamin D levels are associated with higher risk for CVD and more active SLE at baseline.	[403, 407, 408]

25(OH) vit D: 25-hydroxy vitamin D; ACLA: anticardiolipins antibodies; ACR: American College of Rheumatology; anti-*β*2GPI: anti-beta 2 glycoprotein 1 antibodies; aPT: antiprothrombin antibodies; APLA: antiphospholipid antibodies; APS: antiphospholipid syndrome; AT: atherosclerosis; BMD: bone mineral density; BMI: body mass index; CAC: coronary artery calcification; CAD: coronary artery disease; cIMT: carotid intima-media thickness; CRP: C-reactive protein; CV: cardiovascular; CVD: cardiovascular disease; ESR: erythrocyte sedimentation rate; GLADEL: Grupo Latino Americano De Estudio de Lupus; HDL: high-density lipoprotein cholesterol; HRT: hormone replacement therapy; IHD: ischemic heart disease; IMT: intima-media thickness; IRF8: interferon regulatory factor 8; LA: Latin America; LDL: low-density lipoprotein cholesterol; LUMINA: Lupus in Minorities: Nature versus Nurture cohort; MetS: metabolic syndrome; MMP: matrix metalloproteinases; NETs: netosis bodies; PDN: prednisolone; RP: Raynaud's phenomenon; TAG: triglycerides; TRAF: tumor necrosis factor receptor-associated factors; T2DM: type 2 diabetes mellitus; SDI: SLE damage index; SLAM: systemic lupus activity measure; SLE: systemic lupus erythematosus; SLEDAI: Systemic Lupus Erythematosus Disease Activity Index; SLICC: Systemic Lupus International Collaborating Clinics score; SDI: SLICC damage index; SNP: single-nucleotide polymorphism; VLDL: very low-density lipoprotein cholesterol; WC: waist circumference.

**Table 3:** Traditional and nontraditional risk factors associated with CVD and APS.

Risk factor	Comment	Reference
Traditional risk factors		
Metabolic syndrome	The most common risk factors are hypertriglyceridemia, low HDL levels, and visceral obesity.	[409, 410]
Hyperlipidemia	High levels of APLA may be a marker for earlier endothelial damage caused by hyperlipidaemia.	[410, 411]
T2DM	It is associated with cardiovascular disease among APS patients. It did not show any difference between APS patients and the general population.	[410, 412]
Smoking	CVD risk factor increases risk of AT.	[410, 412]
Obesity	Increases the risk of insulin resistance and MetS.	[410, 412]
HTN	Increases risk of ischemic events and CVD.	[410, 412]
Sedentary lifestyle	Increases risk of obesity and comorbidities, propending CVD.	[410, 412]

Nontraditional risk factors		
APS per se	Patients with primary APS have a high prevalence of carotid IMT and a decreased lumen diameter. IMT in primary APS may be associated with stroke. Patients with primary APS with IMT must be considered as carriers of atherosclerosis.	[204]

Autoantibodies	(i) ACLA are associated with a higher risk of venous thrombosis and arterial thrombosis. (ii) Lupus anticoagulant is a major risk factor for arterial thrombotic events. (iii) Immunoinflammatory mechanisms, primarily APLA, have an outstanding role in APS-related vasculopathies. (iv) Patients having APLA and AT may have greater risk for ischemic events than patients with the same degree of AT but without APLA. (v) *β*2GPI is abundantly present in the atherosclerotic plaque. (vi) Anti-*β*2GPI and ACLA may be involved in CAD and stroke. (vii) CAD and PVD occurred more often in patients with elevated serum levels of IgG or IgM APLA, including ACLA or anti-*β*2GPI.	[145, 186, 204, 413–419]

ACLA: anticardiolipins antibodies; anti-*β*2GPI: anti-*β*2 glycoprotein I antibodies; APLA: antiphospholipid antibodies; APS: antiphospholipid syndrome; AT: atherosclerosis; *β*2GPI: *β*2 glycoprotein I; CAD: coronary artery disease; CVD: cardiovascular disease; HDL: high-density lipoprotein; HTN: hypertension; IMT: intima-media thickness; MetS: metabolic syndrome; PVD: peripheral vascular disease; T2DM: type 2 diabetes mellitus.

**Table 4:** Traditional and nontraditional risk factors associated with CVD and SS.

Risk factor		Comment	Reference
Traditional risk factors			
Dyslipidemia		(i) High prevalence of hyperlipidemia and low HDL are associated with CVD and first-degree heart block. (ii) SS patients showed 1.5-fold higher prevalence of hypertriglyceridemia.	[12, 42–44, 210, 420]
T2DM		It is associated with CV compromise in SS patients.	[210]
Advanced age		Age is a predictor for valve compromise	[45]

Nontraditional risk factors			
	Systemic compromise	Articular, renal, liver, peripheral neuropathy, CNS, joint and gastrointestinal involvement, and parotid enlargement are associated with stroke, IHD and lower flow-mediated vasodilation .	[12, 42, 210]
	Polyautoimmunity	SS patients with APS were significantly associated with APLA in thrombotic events.	[41]
SS-associated	Autoantibodies	(i) SS-A is associated with stroke, IHD, and carotid thickening. (ii) SS-B is related to first-degree heart block, valve compromise, and lower nitrate mediated vasodilation. (iii) APLA and lupus anticoagulant are associated with thrombotic events. (iv) ACLA IgG is associated with arrhythmias (v) RF is related to lower nitrate mediated vasodilation. (vi) Anti-HDL.	[12, 41–43, 210, 211, 420]
	Long duration of disease	Longer duration of the disease is associated with stroke and IHD.	[210, 420]
	Chronic proinflammatory state	Elevated CRP is associated with stroke and IHD	[43, 210]
	Glucocorticoids	(i) Steroid use is associated with stroke and IHD (ii) Patients with GCs showed a higher frequency of HTN, T2DM, and elevated TAG.	[42, 210]

Others	Hematological alterations	(i) Hypogammaglobulinemia, leukopenia, thrombocytopenia, and s-VCAM-1 are associated with thrombotic events and lower nitrate mediated vasodilation. (ii) Low C4 and cryoglobulinemia are predictors for valve injury	[12, 42, 45, 210, 211, 420]

ACLA: anticardiolipins antibodies; anti-HDL: anti-high-density lipoprotein antibodies; APLA: antiphospholipid antibodies; APS: antiphospholipid syndrome; CNS: central nervous system; CRP: c-reactive protein; CV: cardiovascular; CVD: cardiovascular disease; GCs: glucocorticoids; HDL: high-density lipoprotein cholesterol; HTN: hypertension; IHD: ischemic heart disease; RF: rheumatoid factor; SS-A: anti-Ro/SSA antibodies; SS-B: anti-La/SSB antibodies; SS: Sjõgren's syndrome; s-VCAM: soluble vascular cellular adhesion molecules; TAG: triglycerides; T2DM: type 2 diabetes mellitus.

**Table 5:** Traditional and nontraditional risk factors associated with CVD and SSc.

Risk factor	Comments	References
Traditional risk factors		
Dyslipidemia	(i) The alteration of lipid profile has been described, given by the increased levels of LDL and lipoprotein A, which are related to the reduction in the fibrinolysis and thrombotic and coronary events. (ii) Decreased levels of HDL are related to anticentromere antibodies positivity. (iii) There is elevation of TAG, total cholesterol, and LDL and decrease in HDL levels.	[214, 218, 421–424]
T2DM	It is associated with CV events in SSc patients.	[54, 424]
Hypertension	Its prevalence increased with the age, and it is correlated with MI.	[54]
Hyperhomocysteinemia	Increased levels are related to AT and endothelial dysfunction.	[218]

Nontraditional risk factors		
SSc per se	It is an independent risk factor for MI	[54]
Autoantibodies	(i) oxLDL/*β*2GPI and anti-oxLDL/*β*2GPI complex: these are considered proatherogenic. (ii) anti-ox-LDL: higher levels are correlated with AT and thrombosis. (iii) anti-LPL: its presence is related to TAG elevated and AT and CV events. (iv) AECA may also contribute to an increased risk of early AT in SSc (v) Others: anticentromere, anti-HSP65/60, and APLA.	[91, 220, 423, 425–429]
Chronic inflammation	Increase of CRP levels and intercellular adhesion molecule-1 may also contribute to an increased risk of early AT in SSc.	[218, 429]

AECA: anti-endothelial cell antibodies; anti-HSP: anti-heat shock proteins antibodies; anti-LPL: anti-lipoprotein lipase antibodies; an anti-oxLDL/*β*2GPI complex: anti-oxidized low-density lipoprotein/*β*2 glycoprotein I antibodies; APLA: antiphospholipid antibodies; AT: atherosclerosis; CRP: c-reactive protein; CV: cardiovascular; CVD: cardiovascular disease; HDL: high-density lipoprotein cholesterol; LDL: low-density lipoprotein; oxLDL/*β*2GPI complex: oxidized low-density lipoprotein/*β*2 glycoprotein I; SSc: systemic sclerosis; TAG: triglycerides; T2DM: type 2 diabetes mellitus.
